# Editorial: Climate change is a children’s health hazard

**DOI:** 10.1088/2752-5309/ad8476

**Published:** 2024-10-24

**Authors:** Caitlin A Gould, Lauren E Gentile, Emily Sbiroli, Martha Berger, Rebecca Philipsborn

**Affiliations:** 1 U.S. Environmental Protection Agency, Office of Research & Development, Center for Public Health and Environmental Assessment, Integrated Climate Sciences Division, Washington, DC, United States of America; 2 U.S. Environmental Protection Agency, Office of Air & Radiation, Office of Atmospheric Protection, Climate Change Division, Climate Science & Impacts Branch, Washington, DC, United States of America; 3 Emergency Department, University of California-San Diego Health, San Diego, CA, United States of America; 4 University of Colorado Anschutz Medical Campus, University of Colorado Climate and Health Program, Aurora, CO, United States of America; 5 Climate Connections, Silver Spring, MD, United States of America; 6 Pediatrics, Emory University School of Medicine, Atlanta, GA, United States of America

**Keywords:** children, health, Children’s health, environmental health, climate change

## Abstract

As temperatures defy heat records, it is difficult to ignore the implications of climate change for public health, including impacts on population health more specifically. In short, climate change is happening now and presents an immediate hazard to human health on a global scale. Age-related health effects are an inalienable truth; physiology is relatively universal, and so are the ways in which our bodies respond to different types and levels of exposures to environmental stressors at different lifestages. Children are uniquely vulnerable to climate change stressors not only due to their physical and developmental immaturity, but also because they generally rely on adult caretakers for the fundamentals of survival. This article is the summary piece accompanying a special issue of Environmental Research: Health. It compiles new studies on children’s vulnerability to climate change as well as studies exploring climate adaptation strategies to promote and protect child health. In this special issue, we see how these concepts are reflected repeatedly in empirical data domestically and internationally. For example, the special issue includes articles investigating linkages between climate change and health hazards such as asthma, injuries, and malnutrition. While local context is extremely important, many of the health effects may be extrapolated to other communities around the world.

As temperatures defy heat records, it is difficult to ignore the implications of climate change for public health, including impacts on population health more specifically. In short, climate change is happening now and presents an immediate hazard to human health on a global scale.

Age-related health effects are an inalienable truth; physiology is relatively universal, and so are the ways in which our bodies respond to different types and levels of exposures to environmental stressors at different lifestages. Children are uniquely vulnerable to climate change stressors not only due to their physical and developmental immaturity, but also because they generally rely on adult caretakers for the fundamentals of survival. For instance, a child’s developing lungs are more sensitive to the inflammatory effects of poor air quality and PM2.5 exposure. Children exposed to pollution have an increased risk of developing chronic diseases such as asthma or chronic obstructive pulmonary disease later in life. Extreme heat causes heat exhaustion and heat stroke, but more specific to children, can undermine attention, cognition, and sleep that are essential to strong performance in school. While infants are exquisitely vulnerable, extreme heat may prove fatal to children regardless of age, as we see each summer with reports of children left in cars or suffering heat stroke during sports events. Stress or trauma from experiencing extreme weather events or fear of housing or food insecurity may manifest as anxiety and depression in children. These impacts are compounded by social determinants of health, environmental injustice, or other factors such as the COVID-19 global pandemic.

A 2023 report by the United States (U S) Environmental Protection Agency detailed ways in which climate stressors are likely to continue to affect children’s health in the contiguous U S throughout this century. Notably, greater rates of new diagnoses of asthma and other respiratory diseases, lost school days and wages due to exposures to poor air quality or high temperatures, greater likelihoods of being diagnosed with Lyme disease, and experiencing partial or total home loss due to coastal flooding were all identified as risks to children (EPA 2023). While the amount of evidence-based research on climate and health continues to grow, children’s needs remain understudied and warrant special consideration.

This special issue compiles new studies on children’s vulnerability to climate change as well as studies exploring climate adaptation strategies to promote and protect child health. In this special issue, we see how these concepts are reflected repeatedly in empirical data in the U S and internationally. For example, the special issue includes articles investigating linkages between climate change and health hazards such as asthma, injuries, and malnutrition. While local context is extremely important, many of the health effects may be extrapolated to other communities around the world.

Helldén *et al* (2023) emphasize the point that lifelong health begins in childhood, urging that climate adaptation efforts must take into account the varied physiological and psychological needs of children across ages and during fetal development. The authors point to common barriers to climate adaptation that can affect children’s health outcomes, many of which stem from the policy process and the efficiency and level (or lack thereof) of public communication and support. In order to develop more effective means of protecting children against the hazards of climate change, the authors encourage policymakers and practitioners to engage children of different ages and to consider their needs throughout design and implementation of forthcoming endeavors.

These sentiments are reflected in ‘Inclusion of child-relevant data in the development and validation of heat vulnerability indices (HVI): a commentary,’ by Weinburger *et al* (2023). Similar to Helldén, they discuss the importance of age and lifestage as key modulators of vulnerability to heat. Weinberger *et al* (2023) assert that HVI are important measures for ascertaining the risk of heat on certain populations’ general health; but also, that age-related characteristics are not always considered during the development of HVIs. Consequently, HVIs that are neither designated specifically for children of different ages nor inclusive of age-related variables outright cannot optimally protect children’s health. The authors note that age-related validation is an important step that should be incorporated into the HVI process. The efficacy and effectiveness of HVIs can be validated against groups of similarly aged children and improve the science community’s fuller understanding of heat impacts on children’s health.

Another paper in this special issue—Girma *et al* (2023)—provides some insight into the importance of factoring in age-related interventions as a part of climate and child-health protection strategies. The case-crossover study conducted by Girma *et al* (2023) shows relationships between exposures to high temperatures, and intentional (e.g. non-accidental) and unintentional (e.g. accidental) injury, with gender and age. Interestingly, they show strong effects during hot weather of school-age males seeking emergency medical attention for unintended injury. In contrast, young-adult males (20–25 years of age) had the highest rates as an age group for experiencing intentional injury during periods of warmer temperatures. Young-adult females had lower rates of injury than their male counterparts but had a greater likelihood of seeking emergency medical attention. These data are fascinating, and inferences from this paper may inform youth violence prevention and risk assessment and abatement efforts.

Similarly, Iyaz *et al* (2023) show in a case-crossover study based on data from Seattle Children’s Hospital from 2006 to 2020 that wildfire smoke days are associated with 9% greater odds of pediatric patient emergency department (ED) visits for respiratory concerns and 11% greater odds of respiratory infections leading to ED visits, compared to non-wildfire days (e.g. the reference period). Additionally, the majority of excess ED visits and hospital admissions in the study are tied to the youngest age group of children considered (0–5 years of age), and, separately, to non-Hispanic Black patients. The latter group in this study shows a 30% greater odds of hospital admissions relative to non-smoke days, which is the greatest increase in odds among the races and ethnicities considered in this analysis. Their work underscores that climate adaptation efforts to protect children must consider not only age but also environmental justice factors that influence exposure and subsequent risk of illness.

Iyaz *et al* (2023) and Girma *et al* (2023) connect well to Avula *et al* (2023), who acknowledge connections between a child’s risk of developing asthma and either a singular or tandem exposure to extreme weather events, including high heat and severe storms. As discussed in this article, social determinants of health and racial or ethnic status compound a child’s likelihood of developing asthma—factors that Girma *et al* (2023) were unable to incorporate in their analysis. Avula *et al* (2023) propose a combination of efforts to limit pediatric asthma development: implementing early interventions to determine a child’s physiological vulnerability for developing asthma and establishing mitigating factors within a child’s built and social environments that improve air quality and ambient indoor temperature.

Wildfire, extreme weather, poor air quality, and heat are visible manifestations of climate change in the U S; however, in other, developing nations, these may be exacerbated by—or may exacerbate—other key child health indicators and outcomes such as undernutrition, burden of infectious diseases, and even mortality. Scarpa *et al* (2023) and Khan *et al* (2023) show linkages between food security and undernutrition status and climate change. The latter paper, ‘Unveiling the link between rainfall, temperature, and childhood undernutrition in Bangladesh using spatial analysis’, applies a spatial mixed-effects logistic regression model to show that greater rates of precipitation and temperatures are linked to childhood undernutrition within Bangladesh. Interestingly, the researchers find alternately positive and negative relationships between extreme rates of rainfall and undernutrition, through direct (e.g. flooding, impacts to food production and distribution) and indirect (e.g. exposure to waterborne diarrhetic pathogens, job loss) forces. Conversely, the team shows that within their study, higher temperatures have a positive relationship with rates of undernutrition. They further note factors that influence the spatial distribution of undernutrition, including climatic factors, rurality, infrastructure availability, and socioeconomic status. Additionally, Scarpa *et al* (2023) elaborates on the tenuous boundary between food security and insecurity, and how factors such as climate change and compounding shocks from the COVID-19 pandemic are tipping points for children and families on the edge. The authors report that food security within many of the southwestern Ugandan households that participated in this study is dependent not only upon successful harvest, but also upon food availability at market—both of which can be highly sensitive and variable. When food is sourced primarily from agrarian sources, per this study, individuals are at greater likelihood of experiencing undernutrition and subsequent health effects. Undernutrition can have the added effect of resulting in more severe sequelae following infection by COVID-19 or other respiratory illnesses.

The literature covered through this special issue provides information on a breadth of concepts linking children’s health and climate change. It is clear from these articles that there are myriad ways in which children may be physically and psychologically impacted by climate change, and that specific attention should be paid to the unique vulnerabilities of each age group during adaptation planning. These important data in this compilation of focused studies shine a light on the path forward to protecting children in the U S and abroad as climate change takes hold.
